# Reverse Osmosis Concentrate: Physicochemical Characteristics, Environmental Impact, and Technologies

**DOI:** 10.3390/membranes11100753

**Published:** 2021-09-30

**Authors:** Hugo Valdés, Aldo Saavedra, Marcos Flores, Ismael Vera-Puerto, Hector Aviña, Marisol Belmonte

**Affiliations:** 1 Centro de Innovación en Ingeniería Aplicada (CIIA), Departamento de Computación e Industrias, Facultad de Ciencias de la Ingeniería, Universidad Católica del Maule (UCM), Av. San Miguel 3605, Talca 3460000, Chile; 2 Departamento de Ingeniería Química, Facultad de Ingeniería, Universidad de Santiago de Chile (USACH), Av. Libertador Bernardo O’Higgins 3363, Estación Central 9160000, Chile; 3 Departamento de Ciencias Básicas, Facultad de Ciencias, Universidad Santo Tomás, Avenida Carlos Schorr 255, Talca 3473620, Chile; marcosflores@santotomas.cl; 4 Centro de Innovación en Ingeniería Aplicada (CIIA), Departamento de Obras Civiles, Facultad de Ciencias de la Ingeniería, Universidad Católica del Maule, Av. San Miguel 3605, Talca 3460000, Chile; ivera@ucm.cl; 5 iiDEA Group, Department of Industrial and Environmental Process Engineering, Engineering Institute, National Autonomous University of Mexico (UNAM), Ciudad de México 04510, Mexico; HAvinaJ@iingen.unam.mx; 6 Laboratorio de Biotecnología, Medio Ambiente e Ingeniería (LABMAI), Facultad de Ingeniería, Universidad de Playa Ancha, Avda. Leopoldo Carvallo 270, Valparaíso 2340000, Chile; marisol.belmonte@upla.cl

**Keywords:** brine, desalination, reject, environmental problems, reverse osmosis, processes

## Abstract

This study’s aim is to generate a complete profile of reverse osmosis concentrate (ROC), including physicochemical characteristics, environmental impact, and technologies for ROC treatment, alongside element recovery with potential valorization. A systematic literature review was used to compile and analyze scientific information about ROC, and systematic identification and evaluation of the data/evidence in the articles were conducted using the methodological principles of grounded data theory. The literature analysis revealed that two actions are imperative: (1) countries should impose strict regulations to avoid the contamination of receiving water bodies and (2) desalination plants should apply circular economies. Currently, synergizing conventional and emerging technologies is the most efficient method to mitigate the environmental impact of desalination processes. However, constructed wetlands are an emerging technology that promise to be a viable multi-benefit solution, as they can provide simultaneous treatment of nutrients, metals, and trace organic contaminants at a relatively low cost, and are socially accepted; therefore, they are a sustainable solution.

## 1. Introduction

Water use has been increasing worldwide by approximately 1% per year since the 1980s. This consumption is divided into three sectors: agriculture (69%), industry (19%), and household (12%) [1]. Globally, the World Water Assessment Programme (WWAP) [2] states that 4000 km^3^/year of drinking water is produced, and, in turn, the International Desalination Association (IDA) [3] reports the existence of a 0.1 km^3^/day cumulative installed desal capacity contributing as a new water source. However, the sources of freshwater for human consumption ordered by level of importance are as follows: surface water > groundwater > rainfall > desalinized seawater > reclaimed wastewater. In all cases, disinfection treatment is required using chlorine, ozone, ultraviolet rays, or other mechanisms that eliminate microorganisms.

**Figure 1:**
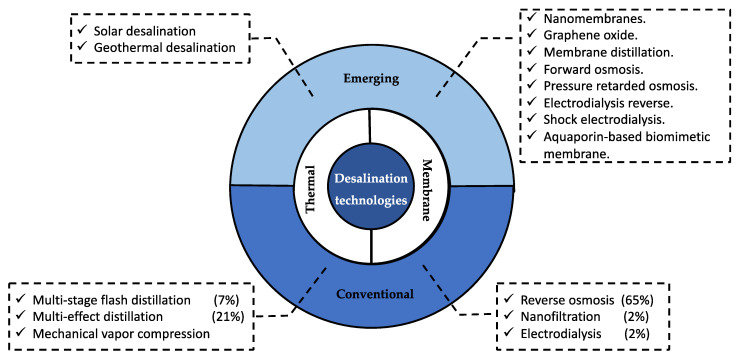
Schematic classification of desalination technologies.

The International Water Association (IWA) [4] stated that desalination technologies are one of three solutions to water scarcity, as these technologies have proven their value, and increasingly use much less energy. Desalination plants have been built mostly in areas where there is a shortage or absence of fresh water, relying on the use of available energy sources at the lowest possible cost [5]. The main desalinated water-producing countries that have more than 5 million m^3^/day installed desal capacity are the Kingdom of Saudi Arabia, United Arab Emirates, United States, Spain, and the People’s Republic of China [3,6,7]. Water for human consumption can be obtained from seawater or brackish water via the desalination process [8,9]. The main desalination technologies are classified into two groups: thermal and membranes [10,11]. Figure 1 shows the conventional and emerging technologies for desalination, highlighting reverse osmosis (RO) and multi-effect distillation (MED) with 65% and 21% of installed capacity worldwide, respectively [12]. RO dominates the global desalination market [13] due to its systematic decrease in energy consumption, from 20 kWh/m^3^ of purified water in the 1970s to 2.5 kWh/m^3^ of purified water today [8,14]. However, several studies have indicated that the main negative impact of this process is brine generation (reverse osmosis concentrate) as a byproduct [15,16,17,18].

Reverse osmosis concentrate (ROC) is a brine that causes several environmental impacts associated with discharge into receiving water bodies [19,20]. In the literature, the ROC effluent is also known as reject [21,22] since the main objective of the desalination process is to obtain water for human consumption. However, currently, there is a growing interest in researching the potential of reusing ROC. For example, Lee et al. [23] demonstrated that ROC usage for brick production satisfied the Korean Standards F 4004 and toxicity characteristic leaching procedure; thus, it is recommended that ROC is used as mixing water to produce calcio sulfoaluminate cement bricks for use in construction. Rana et al. [24] determined that ROC represents a non-toxic, cost-effective, and nutrient-rich growth media for algae cultivation. Jeppesen et al. [25] showed that sodium chloride recovery from ROC can significantly lower the cost of potable water production if synergized with thermal processing systems. Additionally, rubidium recovery from seawater may be a potential source of revenue, and the removal of phosphorus from ROC provides little economic benefit. However, ROC contains considerable amounts of organic phosphonates used as antiscalants and complexing agents, which are regularly discharged into receiving water bodies, thereby posing a risk of eutrophication due to their photolytic and catalytic degradation to bioavailable orthophosphate or accumulation in river sediments, with uncertain long-term consequences. Thus, removing these P-containing organophosphonate compounds from ROC before its further treatment or discharge is imperative [26]. Scholes et al. [27] revealed that open-water wetlands can remove nitrate from RO concentrate at the pilot scale and identified opportunities to enhance treatment efficiency with low-cost carbon amendments.

The purpose of this research is to produce a complete profile of ROC, including physicochemical characteristics, environmental impact, and technologies for ROC treatment, alongside element recovery with potential valorization.

## 2. Methodology

**Figure 2:**
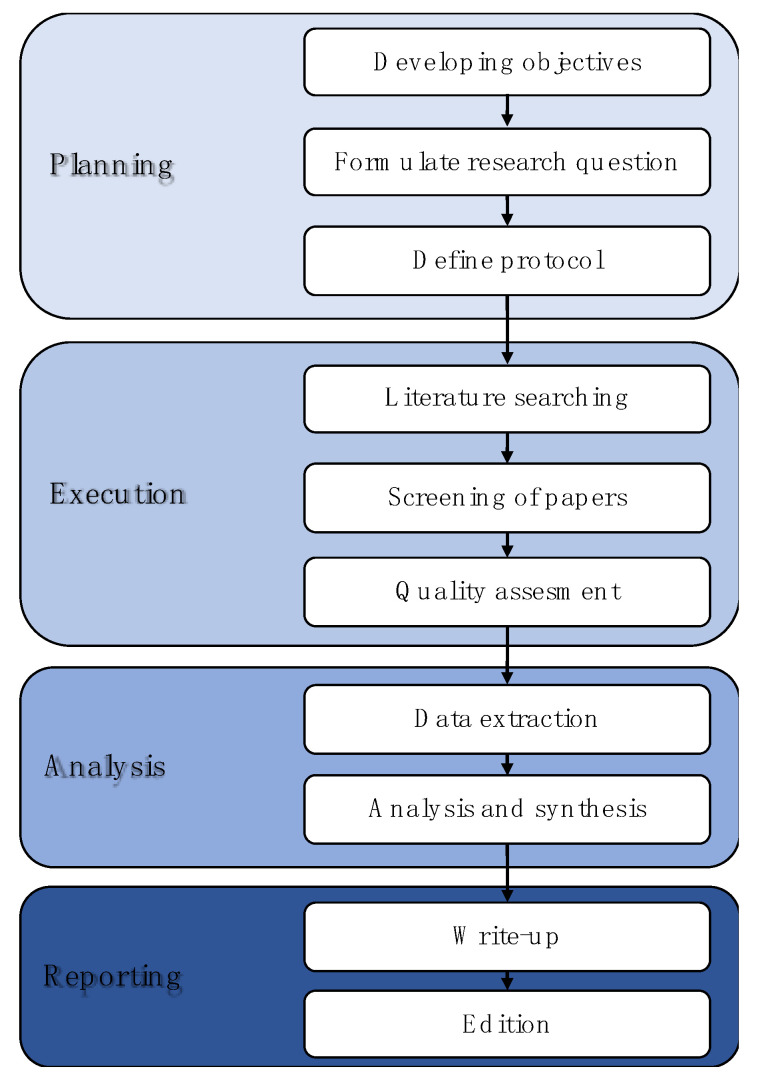
Scheme of SLR stages followed in this research.

A systematic literature review (SLR) was used to compile and analyze scientific information [28,29] about ROC. Figure 2 summarizes the SLR process for this article based on the stages recommended by several authors [30,31,32].

The planning stage began by defining the SLR’s objective: to identify physicochemical characteristics, environmental impacts (effects, regulation, and mitigation), and technologies (conventional and emerging) of ROC. Subsequently, the research questions (RQ) were formulated following the provisions of the PICo (Population, Phenomenon of Interest and Context) elements for qualitative reviews. These PICo elements aid in defining the questions and inclusion criteria used to select studies for systematic reviews [33]. The research questions were as follows:

RQ1: What characteristics of ROC generate an environmental impact on the receiving water body?
RQ2: What technologies mitigate the environmental impact of ROC and provide a revalue to this by product of desalination?

Subsequently, a search and evaluation protocol for the information is established to answer the RQs and achieve the research objective. To address the question of article quality, we decided to include the contents of peer-reviewed journals from WoS and Scopus. This is for the period 2008–2021, including some relevant information published before this period. Table 1 shows the terms (or keywords) used in the search (Title/Abstract/keyword) and the results obtained up to 31 July 2021.

The execution phase started with the literature search in the selected databases (see Table 1). Duplicate articles (present in different databases and combinations) were considered only once for the analysis. Each selected article was categorized as relevant or irrelevant according to the capacity of its title and abstract to answer the research questions of this study. Once the relevant articles were identified, the “Quality assessment” activity was conducted. In this activity, the authors conducted an exhaustive review of the relevant articles to select those closely related to ROC. As in the previous stage, a cross-check of the relevant data found was performed [34].

The analysis stage began with the “data extraction” activity, including obtaining information directly related to the objective of this research. The systematic identification and evaluation of the data/evidence in the articles was conducted with the methodological principles of grounded data theory (GDT) [35]. Through comparisons of the articles, evidence was collected, coded, and analyzed to generate concepts and categories to discover the relationships between these articles and, hence, find decisive evidence for the questions posed and construct explanations for them [35].

Finally, the reporting stage began with a “write-up” activity, including qualitatively integrating the data from the studies by systematically describing them in discussions, figures, and tables. The information analyzed was reported in three main subjects that included several topics about ROC: (A) physicochemical characteristics of ROC; (B) environmental impact of ROC, which includes (1) regulations related to ROC and (2) mitigation and control strategies; and (C) technologies for ROC treatment, which includes (1) conventional technologies and (2) emerging technologies.

## 3. Results and Discussion

**Figure 3:**
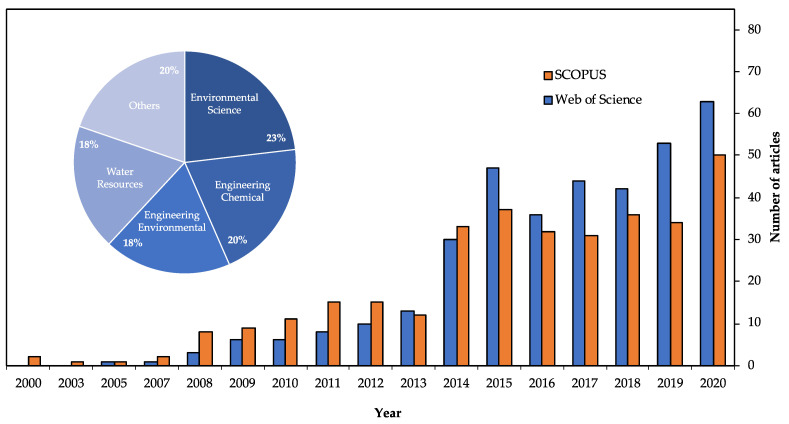
Graphic of articles (topic: “reverse osmosis concentrate”) from 2000 to 2020 in the Web of Science (WoS), SCOPUS, and WoS categories.

SLRs enable us to locate, appraise, and synthesize the best available evidence relating to a specific research question in order to provide informative and evidence-based answers [28]. Figure 3 shows an exponential increase in the number of articles associated with this topic, from two publications in 2000 to more than 50 articles published during 2020. However, the GDT allows for a synthesized description of the research about ROC.

### 3.1. Physicochemical Characteristics of the ROC

The characteristics of ROC depend on several factors, such as the desalination feedwater, membrane type employed, process parameters (i.e., recovery and concentration factor) and additional chemicals used in the pretreatment stage [36,37]. The chemicals used in the feedwater pretreatment stage (membrane desalination) usually include chemicals such as acids, biocides, biocide scavengers, antiscalants, antifoams, and corrosion inhibitors, which can affect the physicochemical composition of the ROC [15,38]. However, environmental conditions (i.e., temperature, pH, and ionic strength) can affect the levels of contaminants present in desalination brine [39]. For instance, among N-nitrosamines (disinfection byproduct), feed solution temperature significantly influences the rejection of N-nitrosodimethylamine [40]. Desalination brine quality also depends on the membrane pore size that is used in the process [39]. Bruggen et al. [41] proposed that small organics and high ion concentrations are present in nanofiltration and ROC. Table 2 shows the main composition of physicochemical parameters present in ROC from desalination plants (seawater or brackish). Some authors have shown the structural characteristics of brine components using FTIR, SEM, and TEM [42,43,44]. Furthermore, Sanmartino et al. [45] identified the phases present in the used RO brine by semi-quantitative analysis of the characteristic peaks obtained for X-ray diffraction, such as NaCl (67%), MgCl_2_·6H_2_O (15%), CaSO_4_ (10%), Mg_3_(SO_4_)_2_(OH)_2_ (3%), Na_2_SO_4_ (3%), and CaMg(CO_3_)_2_ (2%).

### 3.2. Environmental Impact of ROC

ROC is mainly discharged into natural water bodies with or without dilution, depending on the current local environmental regulations and its level of restriction, to avoid degrading marine aquatic ecosystems and the environment [70]. Consequently, several studies have evaluated the environmental impacts of ROC discharge [47,49,71,72]. For example, Dolnicar and Schäfer [73] compared the environmental impact of desalinated water and recycled water and revealed that brine discharge can cause different problems to the environment, such as the destruction of large areas of the ocean floor and severely impacts on the regional environment [70]. In fact, Elsaid et al. [15] showed that emerging desalination technologies focus on maximizing recovery while minimizing energy consumption and cost, alongside applicability to various feed sources while limitedly considering potential environmental impacts. The main causes of the environmental impact of ROC, found in this SLR, are plant outfall, salinity, temperature, pH, chemical products, and heavy metals, where plant outfall has a critical environmental impact as it has direct contact with the marine environment [15]. The main environmental impacts associated with outfalls are biofilm formation, changes in seafloor bathymetry, changes in water circulation patterns, changes in local habitat, and changes in sediment transport patterns [74]. Other impacts are related to ROC composition regarding [75] salinity, temperature, pH, residual chemicals, reaction byproducts, and heavy metals:

○ Salinity and temperature are the major parameters that impact the marine environment, as brine salinity can go up to 65,000–85,000 mg/L (twice the regular seawater concentration), and temperature up to 45–50 °C [76,77,78]. Changes in biota (mainly in plankton and fish species, and pelagic microbial communities) and water quality can occur in the ROC discharge area due to great variations in salinity and temperature [79,80]. These changes are concentrated in the water column and near the seabed, both associated with the discharge point [47].
○ The load of chemical products used during pretreatment as biocides and biocide scavengers, alongside the by-products of the disinfection process, can present ecotoxicity in the marine environment [81,82]. The disinfection by-products (DBPs), upon reaction with natural organic matter present in feedwater, have some ecotoxic effects on aquatic life [83,84,85]. Antiscalant is added to control scaling due to poorly soluble salts, hence maintaining plant productivity—especially at increased recovery [86]. Antiscalants have relatively low toxicity and their environmental fate is defined by their dilution, which further reduces any risk of negative effects; however, their poor degradability is a major drawback [26,87]. Coagulants such as aluminum sulfate, ferric chloride and flocculants are added during pretreatment to enhance the removal of suspended and very fine particles, ending with a filter wash that is disposed of into the brine stream [14] containing iron and aluminum salts with large particles from coagulation and flocculation, which induce some coloring and turbidity effects in receiving waters [88].

ROC contains traces of heavy metals, such as copper, chromium, nickel, iron, and molybdenum, among other elements, as corrosion products of metals by high feedwater salinity [89].

Ramasamy [90] argued that discharging ROC into the sea/ocean causes a “sea desert” in the vicinity of the pipe outlet because the dissolved substance has a high specific weight and thus sinks to the bottom of the sea/ocean, severely affecting the local marine biota, e.g., grass prairies known as *Cymodocea nodosa* and *Caulerpa prolifera* or red algae [91]. Thus, a solution to the above is to dump ROC into the land, but the direct land disposal of ROC causes soil and groundwater contamination by the diffusion of inorganic impurities from it; thus, the soil and ground water are made unsuitable for human consumption due to the presence of toxic substances. It is clear that the environmental impacts of ROC discharges have to be reduced by the introduction of regulations.

To minimize the environmental impacts associated with ROC discharge into the sea, actions are required that aim to (1) establish standards to prevent environmental problems, (2) define mitigation and control strategies, (3) evaluate the potential recovery of ROC (i.e., minerals), and (4) apply treatment technologies.

#### 3.2.1. Regulations Related to ROC

Cornejo et al. [92] revealed that various countries regulate discharge from industrial processes or sewers to ground, surface, or marine water bodies. However, these countries do not reference maximum emission values for the main component of brine, NaCl [93,94]. The “desalination countries” (that have many desalination plants) have their own regulations or multilateral regulations for brine discharge; for example, the Barcelona Convention Protocol in 1976 (modified in 1995) regulates desalination plants of 17 coastal countries in the European Mediterranean Sea. However, “non-desalination countries” are challenged to adapt their effluent discharge regulations, as brine is not usually considered a pollutant.

The Kingdom of Saudi Arabia has the largest installed water production capacity, at 12 Mm^3^/day, representing 9.81% of worldwide capacity, followed by the United Arab Emirates, the United States of America, Spain, and China, at 7.5, 4.7, 3.6, and 3.0%, respectively [18]. The regulations of the Kingdom of Saudi Arabia and the United States require a salinity limit for liquid waste discharge [95,96,97,98]. The United States requires that discharges shall not exceed a daily maximum of 2.0 ppt above natural background salinity (35 ppt) [98], while the Kingdom of Saudi Arabia requires 0.5 mg/L as the maximum limit for chlorine (residual) [96]. Meanwhile, Spain requires an environmental assessment for projects with desalination facilities (volume exceeding 3000 cm^3^/day) [99]. China has focused on regulations and standards to encourage desalination technology utilization, such that seawater utilization was incorporated as an important issue into some formal archives by the end of 2015 [100].

The requirements present in the current regulations applied to the desalination industry are incomplete as countries tend to underestimate the environmental impact that brine generates on the receiving water body.

#### 3.2.2. Mitigation and Control Strategies

**Figure 4:**
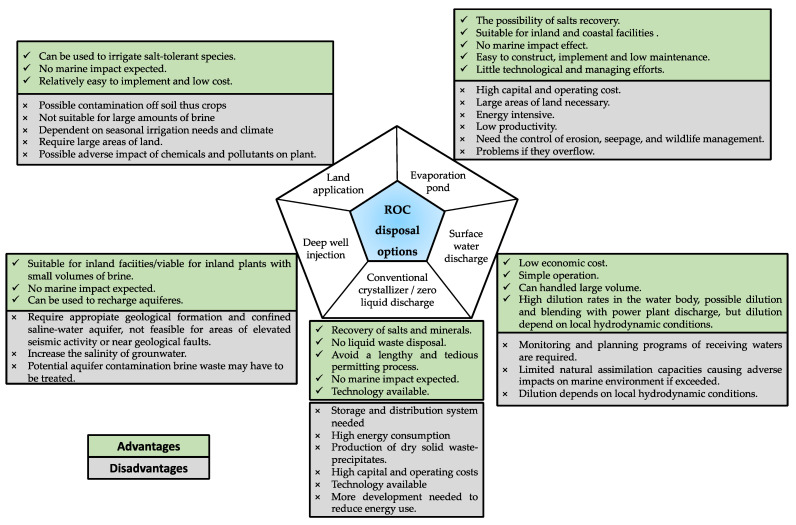
ROC disposal options conceptual scheme.

Disposal options and valorization are two strategies for mitigating the environmental impact of ROC. Figure 4 shows the conceptual scheme for ROC disposal options. Factors affecting the appropriate disposal options of ROC are quality, volume, physical and geographical locations of the output point of the concentrate, economic aspects, social acceptance, authorization of the option, availability of the disposal site, and the feasibility of facility development [18,101]. However, one of the most important factors to be considered before selecting an option is the cost of brine disposal, which impedes the extended use of this process [101]. Hence, emerging technologies consider the circular economy of the process, for example, the production of hydrochloric acid and caustic soda by electrodialysis [72], or the obtaining of salts such as Anhydrite (CaSO_4_), Bischofite (MgCl_2_*6H_2_O), Calcite (CaCO_3_), Carnalite (MgCl_2_*KCl*6H_2_O), Dolomite (CaMg(CO_3_)_2_), Epsomite (MgSO_4_*7H_2_O), Gypsum (CaSO_4_*2H_2_O), Halite (NaCl), Hexahydrite (MgSO_4_*6H_2_O), Kieserite (MgSO_4_*H_2_O), Langbeinite (K_2_SO_4_*2MgSO_4_), Mirabilite (K_2_SO_4_*10H_2_O) + NaCl), Silvite (KCl), and Thenardite (Na_2_SO_4_) by evaporation–crystallization [102]. The advantages and disadvantages of emerging technologies are detailed in Item 3.4.2.

If there is enough aquifer, subsurface intakes provide many environmental benefits and lessen the pretreatment requirements of feedwater, since these intakes achieve full algae and organic biopolymer removal, bacteria removal, and a substantial reduction in transparent exopolymer particles [103]. This substantially reduces desalination costs and energy requirements, making subsurface a green choice for seawater reverse osmosis (SWRO) desalination plants [104,105]. However, subsurface intake is only possible at low possible volumetric flowrates, making it a viable option for small-scale desalination plants (<1000 m^3^/day) [15,49].

Outfall type and design play a critical role in mitigating the environmental impact of brine disposal. Open outfalls are easier and cheaper to construct, with lower operation and maintenance costs. The common outfall for cogeneration plants and wastewater treatment plants helps to reduce its environmental impact by flow equalization and neutralization to uniform salinity and temperature [49]. However, the infrastructure of the outfalls must be of great magnitude to mitigate the effect of brine on the marine environment. Hence, the installation of a diffuser at the end of a 5 km long and 33 m deep pipeline outfall for 65,000 m^3^/day SWRO has been shown to significantly enhance brine mixing with seawater, dropping the salinity from 49,000 mg/L to 38,500 mg/L at a specific point close to the brine discharge, helping in the recovery of benthic community diversity [106].

Hydrodynamic modeling of brine discharge predicts the diffusion and mixing behavior of discharged brine, helping to obtain the pattern of salinity, temperature, and concentration of different contaminants around the discharge point using either near-field or far-field modeling approaches [107]. According to such hydrodynamic modeling results, recommendations for outfall type and design, alongside operation limits regarding salinity and temperature of brine, can be concluded [108].

The elimination of brine in SWRO and brackish water reverse osmosis (BWRO) through the zero liquid discharge (ZLD) process is the ultimate mitigation and control strategy for desalination brine [17,109]. Further, ZLD is a recent technology trend in the desalination process since the Web of Science platform registers an exponential growth for the search “zero liquid discharge” + “desalination”, from three papers in 2010 to 55 papers in 2020. Before 2010, ZLD technologies were considered an uneconomical option and were employed in limited cases [110]. Furthermore, energy is required to drive the brine concentration process (thermal or electrical), and fossil fuels are currently the primary energy source worldwide. ZLD systems capable of managing brine therefore have significant potential for inland desalination in water-scarce regions [111]. However, costs and energy consumption primarily impede full-scale ZLD applications at inland desalination plants in the USA [112].

Currently, the planet is going through an environmental crisis, so the desalination industry cannot be satisfied with the simple discharge of brine but must commit itself to the valorization of brines in different ways (see Table 3). Some elements present in ROC can be extracted and employed in various industrial processes. Elements such as Na, Mg, K, and B can be used in agriculture [113]; Li, In, Rb, Cs, and Ge are suitable for use in the technology industry (batteries, electronic advice, fiber optics, aeronautic, etc.) [114,115,116], while U is used as nuclear fuel for obtaining nuclear energy [117,118]. Hence, Ogunbiyi et al. [119] indicated that sustainable management of ROC is necessary to recover water, energy, and minerals. Otherwise, ROC is discharged directly into water bodies (without treatment), which may cause several environmental impacts [120].

### 3.3. Technologies for ROC Treatment

**Figure 5:**
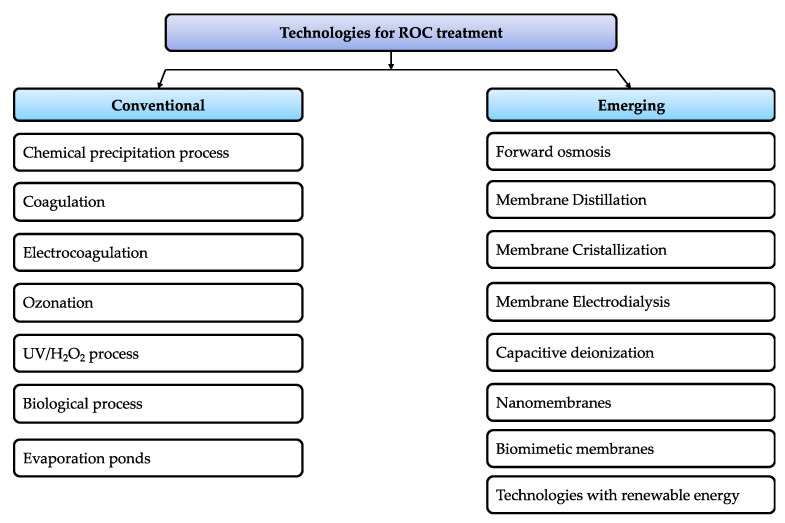
Schematic classification of technologies for ROC treatment.

Technologies for ROC are classified as conventional or emerging, depending on their scientific and technical development level, and their presence in the market (see Figure 5). Based on the definition of emerging desalination technologies proposed by Saavedra et al. [12], the authors propose that emerging technologies for ROC are scientific innovations that generate incentives to invest in ROC treatment. These innovations are based on evolved technologies that improve ROC management strategies (i.e., minimize rejection and/or effluent valorization).

Technologies (or processes) for valorization of ROC are oriented to the recovery of metals that have major difficulties, such as a low concentration of metal ions, limited selectivity of the extracting agents and media used, and the complexity of brine matrices, which severely hamper or rend the process inoperable [59]. Some major elements present at high or relatively high concentrations (e.g., Na, Mg, K, and Br) are currently extracted from seawater and ROC as salts for commercial purposes using very documented processes, e.g., evaporation, precipitation, ion-exchange, solvent extraction, adsorption, membrane separation, etc. [59].

#### 3.3.1. Conventional Technologies

Conventional technologies have focused on removing contaminants from brine before safe disposal for the beneficial use of recovered brine solutions or open water bodies. Chemical precipitation, coagulation, oxidation and biological processes, or combinations thereof, can be used for brine treatment [101]. Several authors have explored the chemical precipitation process for extensive removal of scale-forming ions [58,147,148]. Additionally, coagulation is a basic physicochemical process for charge neutralization and adsorption of organics on metal hydroxides from water and wastewater. This process has been considered for elimination of natural components from highly salted brine arrangements [149,150]. However, electrocoagulation incorporates less slime generation than ordinary coagulation methods [151]. This electrochemical process enables the treatment of highly salted water because it guarantees excellent electric conductivity that might diminish energy utilization [152,153]. Another conventional technology is ozonation, which enables the oxidization of organic compounds (e.g., TOC and COD), either through a coordinated interaction with molecular ozone (O_3_) or through indirect interactions with free radicals (OH^−^). Ozonation has been applied to treat brine, either alone or jointly with other processes [37]. Furthermore, UV/H_2_O_2_ processes can effectively remove organic compounds of various molecular weights present in ROC [154,155]. Finally, high-salinity concentrations result in unbalanced osmotic stress across the microbial cell, affecting the efficiency of biological processes. In addition, the existence of bio-refractory organic compounds in ROC affects the biological process efficiency [156,157].

Evaporation ponds can be extremely expensive; the cost of land procurement for constructing evaporation ponds for ZLD-based desalination schemes in Las Vegas, Nevada, was nearly three times the total cost of brine concentrators and crystallizers. Moreover, water cannot be recovered from evaporation ponds, meaning that there is no additional opportunity to enhance water usage efficiency [109].

#### 3.3.2. Emerging Technologies

Emerging technologies for ROC treatment are focused on improving the results obtained with conventional technologies, reducing the amount of brine to a minimum (or zero), and applying a circular economy.

Most of the technologies are based on membrane separation processes and the use of renewable energies (mainly solar; see Figure 5):

○ Forward osmosis drives water through membranes due to osmotic pressure differences (differences in salt concentration) that are inherently present in the system. Water moves from the feed (low salt concentration) to the draw solution (high salt concentration) [158,159]. The key benefits of using forward osmosis for ROC treatment are: (1) the low energy consumption that comes with it, (2) that high TDS water can be treated, and (3) the lower fouling propensity of the membranes compared to pressure-driven membrane processes [12,160]. However, water flux can be lower than expected in the forward osmosis process due to the existence of internal concentration polarization [161].
○ Membrane distillation is based on the fundamentals of evaporation, and vapor distillate may be produced by temperature, partial pressure, or vacuum gradients [12]. A gas–liquid interface is created as volatile constituents are transferred through a microporous hydrophobic membrane. When water vapor evaporates from the hot brine at the periphery of the brine–membrane interface, it diffuses through hydrophobic membrane pores filled with gas. The water vapor then condenses in the membrane interface at the side, whereby the cooler distillate flows. By heating the feedwater, vapor pressure is increased, thus enhancing the driving gradient for vapor production. The key benefits of using membrane distillation for ROC treatment are: (1) it is operated at low temperatures; (2) it can be retrofitted with heat sources, such as renewable solar energy, geothermal energy, or waste heat sources; and (3) its efficacy is barely affected by the concentration polarization phenomenon, which enables high salt concentrations nearing saturation limits to be fed into the process [162].
○ The benefits of membrane distillation have allowed for the emergence of membrane crystallization (simultaneous production of water and precious crystalline salts) [163,164]. The key benefits of using membrane crystallization for ROC treatment are as follows: (1) higher than average crystallization rates, (2) well-controlled crystal nucleation, and (3) known growth kinetics [165]. Therefore, membrane crystallization is a technology that should be widely addressed in the coming years for ROC treatment.
○ Currently, electrodialysis has been reported to be an efficient method for treating ROC, improving overall RO water recovery to above 90%, and reaching a “near-zero liquid discharge approach” [110,166]. Electrodialysis enables ion transport through an ion exchange membrane using electrical energy as the driving force. These membranes have a high density of ionic groups fixed on them, which allow the selective transport of ions through the membrane depending on their charge. The passage of counter-ions (opposite charge) is allowed, while the passage of co-ions (same charge) is prevented due to Donnan repulsion. Electrodialysis is suitable for ROC treatment since applied electrical energy allows the ions to transfer from the less concentrated solution (water or seawater) to the more concentrated solution (brine). The benefits of using electrodialysis to treat ROC are as follows: (1) low rejection amount, (2) low sensitivity to suspended solids, (3) longer membrane life compared to other applications (e.g., RO), (4) complex pretreatment is not required, (5) ease of operation, and (6) low energy consumption [167,168,169].
○ Another emerging technology related to the electrical charge of the components is capacitive deionization. This technology has received significant attention as an energy-efficient technology for brackish water desalination [170]. Capacitive deionization is an electrochemically induced alternative approach for removing ions from concentrated aqueous solutions by forcing charged ions into the electrical double layer at the electrode–solution interface, where the electrode is connected to an external power supply [55]. The key benefits of using capacitive deionization for ROC treatment are as follows: (1) low operating costs, (2) reduced pretreatment, (3) high recovery, and (4) reduced fouling due to the reversal charge—where the most critical component is that the carbon electrode materials, due to their electrosorptive capacity, depend strongly on physical properties such as the surface area and conductivity of the electrode [22,55].
○ Nanomembranes are membranes that contain nanoparticles (zeolitic type or metal oxide) in the active layer of the polymer matrix, e.g., polymerized polyamide, aiming at improving hydrophilicity, productivity, and salt rejection [171]. Nanomembranes are also known as thin film nanocomposite membranes. Yacou et al. [172] achieved high water fluxes, 10.5 kg/m^2^ h for brackish water at 0.3 wt% salt concentration and up to 4.0–6.0 kg/m^2^ h for 10 wt% salt concentration in reject brine. However, the use of nanomembranes for commercial and industrial RO applications remains underdeveloped, as their scalability remains a challenge [173].
○ Aquaporins are pore-forming proteins in biological cells. Under the right conditions, aquaporin forms a water channel that selectively transports water molecules across while excluding ionic species or other polar molecules. Amy et al. [13] reported that aquaporin-based biomimetic membranes are being developed as ultrahigh permeability RO membranes; with impregnation of aquaporins into a polymeric matrix, aquaporin can provide water channeling/gating, leading to controlled water permeability and ion selectivity [12]. This technology promises high efficiency in ROC treatment since the movement of water in aquaporins is facilitated by “selective rapid diffusion” and an osmotic gradient. The major advantage of aquaporin-based biomimetic membranes is that they don’t require a compromise between selectivity and water permeability. Most applications of aquaporin-based biomimetic membrane technology for water treatment have been conducted using forward osmosis [174].
○ Currently, desalination has high energy demands; hence, integrating renewable energy sources into its process is imperative. However, there are challenges for reducing energy demands and in the use of renewable energy in managing ROC. Okampo and Nwulu [175] explored efficient energy acquirement from renewable energy sources, and brine management in the production of freshwater by synergizing RO, electrodialysis, and crystallization methods. In this case, the brine produced from the RO unit is further desalinated by electrodialysis, leaving a very high concentration to crystallize into soluble salts, thereby achieving a ZLD. The results show that renewable energy sources are more cost-effective and environmentally friendly. Furthermore, the average cost of energy is within the average range of standalone desalination units, suggesting a similar cost of energy for standalone desalination units and combined desalination–brine treatment units.

Most existing ROC treatment technologies are cost prohibitive, but constructed wetlands hold promise as a viable multi-benefit solution as they can provide simultaneous treatment of nutrients, metals, and trace organic contaminants at a relatively low cost. Consequently, some water-stressed cities have already begun experimenting with constructed wetlands for ROC treatment [176,177,178]. However, further research is needed to reduce the land area needed for treatment and to increase the reliability of constructed wetland systems [179].

## 4. Conclusions

The SLR and GDT enabled us to locate, appraise, and synthesize the best available evidence relating to ROC. The value ranges of the physicochemical characteristics of ROC allow us to estimate its chemical composition.

ROC causes various environmental impacts associated with discharge into receiving water bodies, due to its physicochemical characteristics (temperature, pH, salinity, and ions). Furthermore, the requirements present in the current regulations applied to the desalination industry are incomplete since countries tend to underestimate the environmental impact that brine generates in the receiving water body. Therefore, two actions are imperative: (1) countries should generate strict regulations to avoid the contamination of the receiving water body and (2) desalination plants should apply a circular economy.

The literature reviewed indicates that mitigation actions and control strategies are oriented toward ROC valorization, such as energy recovery, metal recovery, and use in constructed wetlands. Hence, a ZLD trend has been driven by the scientific and industrial world due to the need to optimize the use of water resources and to follow environmental regulations (contamination of aquatic environments across the globe). Currently, synergizing conventional and emerging technologies is the most efficient method to mitigate the environmental impact of the desalination process since, traditionally, conventional technologies have focused on removing contaminants from brine before safe disposal for the beneficial use of recovered brine solution or open water bodies, while emerging technologies for ROC treatment focus on improving the results obtained with conventional technologies, reducing the amount of brine to a minimum (or zero), and applying circular economies.

For the coming years, the most promising research and innovation directions for ROC mitigation and/or valorization will be the use of renewable energy (mainly solar energy), nature-based solutions (e.g., constructed wetlands), and nanomembranes. Solar energy is used to generate thermal energy or electrical energy, either for distillation or electrodialysis, respectively. Constructed wetlands are an emerging technology that promises to be a viable multi-benefit solution as they can provide simultaneous treatment of nutrients, metals, and trace organic contaminants at a relatively low cost. In addition, they are socially acceptable; therefore, they are a sustainable solution. Conversely, nanomembranes comprise nanoparticles that attract water and absorb it like a sponge while repelling almost all the pollutants that usually adhere to the membrane surface. Therefore, using nanomembranes, water can be obtained from brine using less energy than a traditional RO.

## Figures and Tables

**Table 1:** Keyword combinations to search for information about ROC.

Keywords	K1: Reverse Osmosis Concentrate K2: Reverse Osmosis Reject K3: Reverse Osmosis Brine K4: Component K5: Environmental Impact	K6: Regulation K7: Recovery K8: Treatment K9: Management K10: Zero Liquid Discharge	
Combinations for Search in TITLE-ABS-KEY		Number of Articles	
		WoS	Scopus
C1: K1		389	351
C2: K2		30	34
C3: K3		103	107
C4: (K1 OR K2 OR K3) AND K4		24	67
C5: (K1 OR K2 OR K3) AND K5		9	12
C6: (K1 OR K2 OR K3) AND K6		14	12
C7: (K1 OR K2 OR K3) AND K7		158	141
C8: (K1 OR K2 OR K3) AND K7 AND K8		102	95
C9: (K1 OR K2 OR K3) AND K7 AND K8 AND K9		22	26
C10: (K1 OR K2 OR K3) AND K7 AND K8 AND K9 AND K10		9	4

**Table 2:** Possible range of the main physicochemical parameters present in ROC.

Parameter	Possible Range	Unit	References
pH	6.2–8.2	unit	[45,46,47,48,49]
Temperature	24–28	°C	[50,51,52]
Conductivity	25,000–91,000	µS/cm	[48,53,54,55]
Turbidity	0.45	NTU	[48]
TDS ^(1)^	10,000–70,000	mg/L	[45,46,48,51,53,55,56,57]
Alkalinity ^(2)^	140–1500	mg/L	[45,51,57,58]
TOC ^(3)^	1.5–142	mg/L	[51,54,56]
Na^+^	3300–25,000	mg/L	[45,47,48,51,52,53,54,56,57]
Mg^2+^	200–7600	mg/L	[45,47,48,51,52,53,54,56,57]
K^+^	80–850	mg/L	[46,51,52,53,54,59]
Ca^2+^	87–2800	mg/L	[45,47,48,51,52,53,54,56,57,58]
B	5.0–9.5	mg/L	[55,59]
Li	0.3–0.6	mg/L	[59,60,61]
In	0.02	mg/L	[59,62]
Rb	0.1–0.2	mg/L	[59,62]
Cs	0.0005–0.0008	mg/L	[59,62]
U	0.0039	mg/L	[59,63]
Ge	0.00007	mg/L	[59,62]
Fe^2+^	0.001–0.4	mg/L	[46,55]
Mn^2+^	0.1–0.3	mg/L	[46,64]
Sr^2+^	9–18	mg/L	[55,65,66]
Si	9–11	mg/L	[45,66]
SiO_2_	18–140	mg/L	[47,51,57,66,67]
Cl^−^	6500–42,000	mg/L	[45,51,52,53,54,56,57,66]
Br^−^	90–230	mg/L	[52,54,59]
SO_4_^2−^	1600–8000	mg/L	[45,47,51,52,53,54,56,57,59,65]
NO_3_^−^	1.8–15	mg/L	[46,55,66]
PO_4_^3-^	0.4–2.5	mg/L	[57,68,69]
HCO_3_^−^	140–3900	mg/L	[47,55,56,57,68]
Anionic detergents	112–126	μg/L MBAS	[45]

(1) TDS: Total dissolved solids; (2) mg/L as CaCO_3_; (3) TOC: Total organic carbon.

**Table 3:** List of main ROC valorization strategies (based on [61]).

Type of ROC valorization	Strategies	Reference
Uses inside the desalination plant	Source of water for pretreatment backwash	[121]
	Generation of chlorine via electro-chlorination	[122]
	Production of acids (HCl) and basic compounds (NaOH) through electrodialysis	[123]
As a source of minerals	Evaporation–crystallization (ZLD)	[124]
	Evaporation ponds	[125]
	Desalination plants for combined water and salt production	[126]
	Salt solidification and sequestration	[46]
	Intensive evaporation processes	[127]
	Electrodialysis for salt recovery	[128]
	Ion exchangers for salt recovery	[129]
	Solvent extraction	[59]
	Supercritical water	[130]
	Hybrid processes including RO, NF, and precipitation	[131]
For energy and energy production	Energy recovery	[132]
	Energy production with turbines	[133]
	Energy production using the osmotic potential energy	[134]
	Technologies based on solar ponds	[135]
Environmental applications	Land application	[136]
	Regeneration of degraded areas	[137]
In aquaculture and fish farming	Use of microalgae as biomass for removing certain salts	[138]
	Inland saline aquaculture	[139]
Other potential uses	Agriculture irrigation	[140]
	Hydrotherapy	[141]
	Secondary recovery of oil through deep well injection of brine and/or CO_2_	[142]
	Food industry	[143]
	Growing of halophiles	[144]
	CO_2_ retention technologies	[145]
	Deicing and dust suppression	[146]

## Data Availability

Not applicable.
